# Nitrate nitrogen enhances the efficiency of photoprotection in *Leymus chinensis* under drought stress

**DOI:** 10.3389/fpls.2024.1348925

**Published:** 2024-02-14

**Authors:** Xiaowei Wei, Lin Han, Nan Xu, Mingyue Sun, Xuechen Yang

**Affiliations:** ^1^ Jilin Provincial Key Laboratory for Plant Resources Science and Green Production, Jilin Normal University, Siping, China; ^2^ State Key Laboratory of Black Soils Conservation and Utilization, Northeast Institute of Geography and Agroecology, Chinese Academy of Sciences, Harbin, Heilongjiang, China; ^3^ Key Laboratory of Heilongjiang Province for Cold-Regions Wetlands Ecology and Environment Research, and School of Geography and Tourism, Harbin University, Harbin, China

**Keywords:** ammonium, electron transport, *L. chinensis*, nitrate, photoprotection

## Abstract

**Introduction:**

Global climate change exerts a significant impact on the nitrogen supply and photosynthesis ability in land-based plants. The photosynthetic capacity of dominant grassland species is important if we are to understand carbon cycling under climate change. Drought stress is one of the major factors limiting plant photosynthesis, and nitrogen (N) is an essential nutrient involved in the photosynthetic activity of leaves. The regulatory mechanisms responsible for the effects of ammonium (NH_4_^+^) and nitrate (NO_3_^-^) on the drought-induced photoinhibition of photosystem II (PSII) in plants have yet to be fully elucidated. Therefore, there is a significant need to gain a better understanding of the role of electron transport in the photoinhibition of PSII.

**Methods:**

In the present study, we conducted experiments with normal watering (LD), severe drought (MD), and extreme drought (HD) treatments, along with no nitrogen (N0), ammonium (NH_4_), nitrate (NO_3_), and mixed nitrogen (NH_4_NO_3_) treatments. We analyzed pigment accumulation, reactive oxygen species (ROS) accumulation, photosynthetic enzyme activity, photosystem activity, electron transport, and O-J-I-P kinetics.

**Results:**

Analysis showed that increased nitrate application significantly increased the leaf chlorophyll content per unit area (Chl_area_) and nitrogen content per unit area (N_area_) (p< 0.05). Under HD treatment, ROS levels were lower in NO_3_-treated plants than in N0 plants, and there was no significant difference in photosynthetic enzyme activity between plants treated with NO_3_ and NH_4_NO_3_. Under drought stress, the maximum photochemical efficiency of PSII (Fv/Fm), PSII electron transport rate (ETR), and effective quantum yield of PSII (φPSII) were significant higher in NO_3_-treated plants (p< 0.05). Importantly, the K-band and G-band were higher in NO_3_-treated plants.

**Discussion:**

These results suggest that drought stress hindered the formation of NADPH and ATP in N0 and NH_4_-treated *L. chinensis* plants, thus damaging the donor side of the PSII oxygen-evolving complex (OEC). After applying nitrate, higher photosynthetic enzyme and antioxidant enzyme activity not only protected PSII from photodamage under drought stress but also reduced the rate of damage in PSII during the growth of *L. chinensis* growth under drought stress.

## Introduction

Against the backdrop of global climate change, nitrogen (N) deposition, especially wet deposition, coincides with precipitation events, exacerbating water scarcity during the summer (IPCC, 2021). Drought is expected to pose a threat to the extensive livestock industry on grasslands by affecting the production of forage, particularly in areas with a high biomass growth (Olesen et al., 2011). Indeed, drought not only hinders the water uptake by plants, but it can also severely limit the effectiveness of soil nutrients, particularly soil nitrogen, by restricting mineral nitrogen flux (Gonzalez-Dugo et al., 2010). Apart from affecting nutrient uptake and utilization by plants, drought can also lead to a reduction in carbon assimilation, thus resulting in reduced photosynthetic productivity and the restriction of growth and development (Liang et al., 2022). One crucial reason for the reduced plant growth under drought conditions is the disruption of cellular redox homeostasis (Huang et al., 2021).

Two major forms are involved in the deposition of nitrogen: ammonium nitrogen (NH_4_^+^-N) and nitrate nitrogen (NO_3_^-^-N). However, the effects of the slow deposition of NH_4_^+^-N and NO_3_^-^-N on the growth and primary productivity of plants are known to differ (LeBauer and Treseder, 2008; Xia and Wan, 2008; Fernández-Martínez et al., 2014). Consequently, different nitrogen forms and drought can exert significant effects on the growth of the dominant species, *L. chinensis*, which in turn affects the physiology and morphology of the plants, resulting in constraints on the growth, yield, and quality of the forage (Jump and Peñuelas, 2005). Both NH_4_^+^-N and NO_3_^-^-N can exert important influences on steppe vegetation. The plasticity of different functional traits in *L. chinensis* undergoes significant changes with increasing nitrogen application rates, with the addition of NH_4_NO_3_ promoting stem elongation and increasing plant height, leading to accelerated growth (Bai et al., 2020). The enhanced nitrogen uptake by plants can improve photosynthetic capacity, thereby promoting the growth of leaves and the above-ground biomass (Vries et al., 2016; Prinsi and Espen, 2018). Different forms of nitrogen affect the balance of nitrogen allocation within *L. chinensis* leaves and can regulate the entry of more NO_3_^-^-N into the photosynthetic system to participate in the Calvin cycle, thereby increasing the photosynthetic nitrogen use efficiency (PNUE) (Wei et al., 2022).

The effective management of nitrogen fertilizer can alleviate the impact of drought on plants by maintaining normal physiological regulation and by scavenging the reactive oxygen species (ROS) generated by drought stress (Da Leite et al., 2018; Zhong et al., 2019; Lv et al., 2021). Specifically, variations in nitrogen uptake and utilization can alter nitrogen metabolism and carbon fixation in the photosynthetic system, consequently impacting the formation of cellular structure (Liu et al., 2021; Wei et al., 2022), leading to negative feedback on carbon balance (Gessler et al., 2017). Previous studies have shown that high concentrations of NH_4_^+^-N can associate with the oxygen-evolving complex (OEC), leading to a reduction in PSII quantum efficiency, while high concentrations of NO_3_^-^-N enhance PSII electron transfer efficiency (ETR) (Wei et al., 2022). Previous research has indicated that most species are sensitive to NH_4_^+^-N, but high concentrations of NH_4_^+^-N can lead to various metabolic disruptions (Li et al., 2012; Britto and Kronzucker, 2013). Compared to NO_3_^-^-N conditions, plants exhibit slower growth, increased oxidative stress, and alterations in the mitochondrial and chloroplast metabolism in environments with excessive NH_4_^+^-N (Ariz et al., 2010; Yang et al., 2014). Much of the previous research on NH_4_^+^-N and NO_3_^-^-N has focused on the adaptation mechanisms of plants to NH_4_^+^ toxicity, such as the GS/GOGAT cycle and antioxidant enzyme systems (Balkos et al., 2010).

Plants exposed to abiotic environmental stress conditions, stimulate the formation of ROS in chloroplasts, thus resulting in a reduction in in PSII quantum yield regulated by the xanthophyll cycle and a decline in proton gradient across the thylakoid membrane caused by cyclic electron flow through Photosystem I (PSI) or PSII (Gill et al., 2016). PSII is highly sensitive to environmental changes, and environmental stress is known to inhibit the interaction between electron donors and acceptors, inducing changes in the I-P transition associated with the redox thylakoid potential generated by PSII during the process of electron transfer from PQ or PQH2 to the PSI electron acceptors (Fd and NADP), which regulate proton pumping through cyclic electron transport in PSI (Batra et al., 2014). Compared to PSII, PSI exhibits higher resistance to water deficiency, and only negative effects occur under extreme drought conditions (Souza et al., 2004). Reduced CO_2_ assimilation may lead to an imbalance between PSII photochemical activity and NADPH demand. In such cases, increased production ROS may be responsible for the increased sensitivity of PSII to photodamage (Ohashi et al., 2006). In most cases, chlorophyll fluorescence measurements indicate that enhanced protection of PSI and PSII photochemistry occurs via adjustments in the distribution of energy between the photosystems and activation of alternative electron flows (Alp et al., 2023).

The Songnen grassland, a typical semi-arid grassland, is limited by nitrogen availability; *L. chinensis* is the dominant species of plant in this region (Zhu, 2004; Song et al., 2023). The increased deposition of atmospheric nitrogen driven by climate change affects the N uptake of *L. chinensis*. In semi-arid ecosystems, primary productivity and plant functional traits are co-limited by water and N availability (Meng et al., 2021), thus highlighting the potential regulatory role of water use efficiency in the response of productivity and plant functional traits to N addition. The above-ground growth of *L. chinensis* is a key factor that influences the response of above-ground net primary productivity to rainfall patterns and N deposition in the Songnen grassland. Under future climate change scenarios, the above-ground net primary productivity of the grassland is expected to have some recovery capacity (Shi et al., 2022). Photosynthesis is the main source of energy for above-ground plant biomass, and the efficiency of photosynthesis can directly influence the above-ground productivity of plants. Effective N fertilizer management can mitigate the effects of drought on plants by maintaining normal physiological regulation and by clearing ROS formed by drought stress (Zhong et al., 2019; Lv et al., 2021). However, only limited research has focused on the impact of the photosynthetic electron transport chain on plant response to drought stress under NH_4_^+^-N and NO_3_^-^-N conditions, especially under long-term drought with moderate NH_4_^+^-N or NO_3_^-^-N supplementation.

This study aimed to investigate the regulatory effects of different N forms on the photosynthetic apparatus activity of *L. chinensis* leaves under drought stress. Existing studies mainly focus on comparing the effects of NH_4_^+^-N and NO_3_^-^-N nutrition on photosynthetic parameters in different species, rather than evaluating plant responses based on photosynthetic mechanisms (Ding et al., 2015; Cao et al., 2018). Further research is therefore needed to investigate the interactions between NH_4_^+^-N, NO_3_^-^-N and photosynthetic mechanisms in grasses, particularly in dominant grasses such as ryegrass, which are highly dependent on N sources in grassland ecosystems. Previous studies have yielded conflicting conclusions with regards to the preferential utilization of NH_4_^+^-N or NO_3_^-^-N by *L. chinensis*. Only a few studies have investigated the regulation of plant photoprotection ability by different forms of N to adapt to environmental stress. In the present study, the effects of different forms of N (sole NH_4_^+^, sole NO_3_^–^ and mixed NH_4_^+^/NO_3_^–^: 50%/50%) on leaf plant photoprotection ability were investigated under field conditions to elucidate the physiological mechanism of NO_3_^-^-N assimilation and enzyme regulation in the photosynthetic systems of *L. chinensis* leaves and to enrich our understanding of drought tolerance in the leaves of *L. chinensis*. Our findings led to the generation of three key hypotheses: (1) NO_3_^-^-N enhances antioxidant enzyme activity to facilitate the scavenging of ROS; (2) NH_4_^+^-N treatment may restrict photosynthetic electron transport when compared to NO_3_^-^-N treatment, and (3) drought stress exacerbates the limitation of photosynthetic electron transport, although the inhibition of electron transport was effectively alleviated by NO_3_^-^-N supplementation.

## Materials and methods

The research took place at the Jilin Songnen Grassland Ecosystem National Observation and Research Station within Northeast Normal University in Jilin Province, China, positioned at 44°34’N, 123°31’E. The location is characterized by a semi-arid, semi-humid climate with temperate continental monsoon influences. The region is known for its hot and wet summers, contrasted by cold and dry winters, with mean temperatures ranging from 4.5 to 6.5°C. The annual precipitation varies between 280 to 620 mm, predominantly from June to September, and the average annual rainfall is about 1200 to 1300 mm, as reported by Shi et al. (2021) and Wei et al. (2022). The soil within the upper 20 cm layer exhibits a pH of 8.75, an electrical conductivity of 78.16 μs cm^-1^, and contains 1.14 g kg^-1^ of total N, 0.68 g kg^-1^ of total phosphorus, 6.43 g kg^-1^ of organic carbon, 1.34 mg kg^-1^ of ammonium nitrogen, and 2.51 mg kg^-1^ of nitrate nitrogen.

*Leymus chinensis* (Trin.) Tzvel., a C_3_ rhizomatous perennial grass, is prevalent in northern China, eastern Mongolia, Transbaikalia, and parts of Russia. It is exceptionally resilient to diverse environmental stressors such as drought, salinity, alkalinity, and cold. This adaptability often establishes it as the dominant flora in its preferred habitats of steppes and meadows, as documented by Liu et al. (2019). On April 20th, *L. chinensis* shoots were meticulously transplanted into monoculture plastic pots, with each pot accommodating four plants, and these pots measuring 15 cm in diameter by 25 cm in depth, were filled with aeolian sandy soil, weighing 3.5 kg pot^-1^. To evaluate their growth response, different N concentrations were applied across treatment groups (Wei et al., 2022). On May 1st, 2020, a rain shelter was erected, and *L. chinensis*, grown in 2019, was moved to the pots. Following a 10-day acclimatization, on May 15th, pots were thinned to four seedlings each to promote even growth and create a consistent system for the upcoming fertilizer and drought assessments. The pot trial adhered to a completely randomized block design, featuring six replications per treatment, with each block having four N application schemes and three levels of drought stress (n = 6).

The N treatments included: no fertilization (N0), solely ammonium nitrogen (NH_4_^+^) from (NH_4_)_2_SO_4_ (NH_4_), exclusively nitrate nitrogen (NO_3_^–^) from Ca(NO_3_)_2_ (NO_3_), and an equimolar mix of NH_4_^+^ and NO_3_^–^ using NH_4_NO_3_. Different forms of nitrogen fertilizer were prepared into solutions with a concentration of 10 g N m^–2^. The solution was evenly distributed at 10 points in the pot, and injected into the soil at a depth of 1-2 cm using a disposable syringe on May 20th and June 6th, respectively, with equal amounts of nitrogen solution. Prior studies in northern grasslands have determined the peak N deposition rate to be 10 g N m^–2^y^-1^ (Zhang et al., 2017). The medium with only NH_4_^+^-N was balanced using CaCl_2_ (39.7 g m^–2^). To prevent NH_4_^+^ nitrification, a nitrification inhibitor, dicyandiamide (DCD, 98.0%), was introduced at 10 mg m^–2^ per annum for the NH_4_^+^ treatment and 5 mg m^–2^ per annum for the NH_4_NO_3_ mix. Additional fertilizers and micronutrients were provided across all treatments to eliminate any non-nitrogen nutrient limitations on plant growth. Regarding drought treatments, three soil water content (SWC) levels were designed: control (LD, SWC of 65%-70%), moderate drought (MD, SWC of 45%-50%), and severe drought (HD, SWC of 25%-30%), based on the field capacity. The SWC was managed by the gravimetric method, adjusting the pots’ weight every two days between 3:00 pm and 7:00 pm to maintain the desired SWC. The SWC calculation is: SWC (%) = (W1 - W2)/W2, where W1 is the weight of soil current and W2 the weight of the oven-dry soil. Weed, pest, and disease control was diligently performed throughout the season. Harvest occurred on August 20th, aligning with the post-fruiting growth phase.

### Leaf photosynthesis measurements

From July 15th to 30th, 2020, the leaf assimilation rate (An, μmol m^-2^ s^-1^) was recorded using a CIRAS-3 portable photosynthesis system (PP Systems, USA). The measurements were taken at a stable temperature of 25°C, with a CO_2_ concentration set at 400 μmol mol^-1^, a flow rate of 500 μmol s^-1^, and a photosynthetic photon flux density (PPFD) of 1600 μmol m^-2^ s^-1^ within the leaf chamber. For each plant, the gas exchange in leaves was gauged on the second and third leaves from the shoot apex, with the recordings done between 8:00 am and 4:00 pm, ensuring six replicates for consistency.

### Chlorophyll *a* fluorescence measurement and O-J-I-P transient analyses

From July 15th to 30th, the dynamics of the chlorophyll a O-J-I-P kinetic curve were measured using a Handy-PEA continuous excitation chlorophyll fluorimeter from Hansatech Instruments, adhering to Strasser’s method and conducted at ambient temperatures (Strasser and Govindjee, 1992; Strasser et al., 2000). Initially, leaf samples underwent a 30-minute dark adaptation phase, using clips to avoid the midrib, ensuring uniform darkening. The rapid fluorescence kinetics were tracked from 10 ms to 1 s, with FO, FJ, FI, and FP denoting fluorescence intensities at 20 ms, 2 ms, 30 ms, and 300 ms, respectively. The K peak at 300 ms on the kinetic curve was particularly noted (Strasser, 1997; Strasser et al., 2000; Ronde et al., 2004; Strasser et al., 2004; Li et al., 2009). *PI*_abs_, indicative of energy conservation efficiency from excitation to electron acceptor reduction, was calculated (Strasser et al., 2004; Strauss et al., 2006). A comprehensive fluorescence kinetics analysis was conducted, entailing various normalization and kinetic differential calculations, with key parameters and formulas detailed in **Table 1** (Strasser et al., 2000; Cun et al., 2022) (n = 6).

**Table 1:** Formulae to calculate the technical data of the O-J-I-P curves used in this study.

V_t_ = (F_t_-F_O_)/(F_M_-F_O_)
V_J_ = (F_J_-F_O_)/(F_M_-F_O_)
M_0 = _4(F_270μs_-F_O_)/(F_M_-F_O_)
S_m_ = Area/(F_M_-F_O_)
ψEo = ET_0_/TR_0_=(1-V_J_)
φPo = TR_0_/ABS=1-F_O_/F_M_
φEo = ET_0_/ABS=(1-F_O_/F_M_) (1-V_J_)
γRC = Chl_RC_/Chl_total_=RC/(ABS+RC)
ABS/CS =Chl/CS
TR_0_/CS=φPo×(ABS/CS)
ET_0_/CS =φPo×ψEo×(ABS/CS)
RC/ABS = γRC/(1-γRC) = φPo (V_J_/M_0_)
RC/CS = φPo×(V_J_/M_0_) × (ABS/CS)
Q_A_-reducing centers = (RC/RC_reference_) ×(ABS/ABS_reference_) = ((RC/CS) _treatment/_(RC/CS) _control_) ((ABS/CS) _treatment_/(ABS/CS) _control_)
Q_B_-reducing centers = φPo^*^/φPo = (1-F_O_/F_M_) _(secondexposure)_/(1-F_O_/F_M_)_(firstexposure)_
S_m_/t_Fmax_ =[RC_open_/(RC_close_+RC_open_)] av = [Q_A_/Q_A_ (_total_)] av
R_J_ = (ψEo_(control)_- ψEo_(treament)_)/(ψ_Eo(control)_) = (V_J (treatment)_-V_J (control)_)/(1-V_J (control)_)
${\text{PI}}_{\text{abs}}=\frac{\gamma \text{RC}}{1-\gamma \text{RC}}\times \frac{\varphi \text{Po}}{1-\varphi \text{Po}}\times \frac{\psi \text{Eo}}{1-\psi \text{Eo}}$
${\text{PI}}_{\text{total}}\equiv \frac{\gamma \text{RC}}{1-\gamma \text{RC}}\times \frac{\varphi \text{Po}}{1-\varphi \text{Po}}\times \frac{\psi \text{Eo}}{1-\psi \text{Eo}}\times \frac{\delta \text{Ro}}{1-\delta \text{Ro}}$

Further, the effective quantum yield of PSII (φPSII), the non-photochemical quenching coefficient (NPQ), and the electron transport rate (ETR, μmol e^-1^ s^-1^ m^-2^) were measured using an IMAGING PAM M-series from Walz, with a 30-minute dark acclimation preceding the measurements to assess the PSII quantum efficiency of the plants (n = 6).

### Leaf biomass and nitrogen content

Following the measurement of chlorophyll fluorescence parameters, two leaves from each plant were harvested, instantly frozen in liquid nitrogen, and preserved at -80°C for subsequent biochemical analyses. Another two leaves were heat-fixed at 105°C for 30 minutes and then dehydrated to constant mass at 65°C for the determination of leaf dry weight and leaf mass per unit area (LMA, g m^-2^) (n = 6).

Accurately weigh a certain amount of ground plant samples (2-3 mg) into tin foil cups, tightly wrap them, record the mass, and place them in the automatic sample introduction tray of the instrument for analysis using a stable isotope ratio mass spectrometer (Elementar, Isoprime 100, UK) to determine the N content (N_m_, g/kg) in the leaves, stems, and roots. Leaf N content per unit leaf area (N_area_, g m^-2^) was calculated by N_m_ × LMA (n = 6).

### Leaf photosynthetic pigment analyses

Chlorophyll *a* and chlorophyll *b* concentrations were quantified from 0.1 g of leaf tissue in ethanol extracts, with absorbance readings taken at 645 nm and 663 nm using a UVmini-1240 spectrophotometer (Shimadzu, Japan) (Wellburn, 1994; Wei et al., 2022). The total chlorophyll content per unit area (Chl_area_, mg m^-2^) was then calculated by multiplying chlorophyll *a+b* with the leaf mass per unit area (LMA) (n = 6).

### Determination of reactive oxygen species content

The content of reactive oxygen species (ROS) and hydrogen peroxide (H_2_O_2_), were determined by corresponding ELISA kits (Shanghai Jining Biology Co., Ltd., China). Conduct sample processing according to the instructions of the kits, the concentrations were measured using an enzyme reader (Bio-imark, BIO-RAD, USA) at the corresponding wavelengths (ROS at 450 nm and H_2_O_2_ at 240 nm).

### Leaf photosynthetic enzyme activity

The enzyme activities of ribulose-1,5-bisphosphate carboxylase/oxygenase (Rubisco), the reduced form of nicotinamide adenine dinucleotide phosphate (NADPH), alternative oxidase (AOX), and ascorbate peroxidase (APX) were assessed using specific ELISA kits provided by Shanghai Jining Biology Co., Ltd., China. The sample preparation was executed in strict adherence to the kit protocols. The concentrations were then quantified using an enzyme reader (Bio-imark, BIO-RAD, USA), with absorbance measurements taken at the respective wavelengths for each enzyme (Rubisco at 340 nm, NADPH at 450 nm, AOX at 450 nm, and APX at 290 nm).

### Statistical analysis

Data processing and visual analysis were conducted using R software version 4.0.4 (RStudio, USA, [https://www.r-project.org/]) (R Core Team, 2021). To assess significant differences among treatment groups, the “Fisher’s LSD” function in the “agricolae” package was applied (P< 0.05). For correlation analysis, the “pearson” function in the “gpairs” and “ggpmisc” packages was utilized, and the “ggplot2” package was employed for creating graphics. The statistical tests included one-way ANOVA to evaluate the impact of various N forms and drought stress on plant parameters, and two-way ANOVA for investigating the effects of nitrogen forms (N), drought stress (D), and their interaction. The Pearson correlation coefficient was used to explore relationships between different plant parameters. All datasets were initially tested for normal distribution using the Kolmogorov-Smirnov test and for variance homogeneity using Levene’s test.

## Results

### Effects of different N forms on Chl content, nitrogen content and photosynthetic rate under drought stress

**Figure 1:**
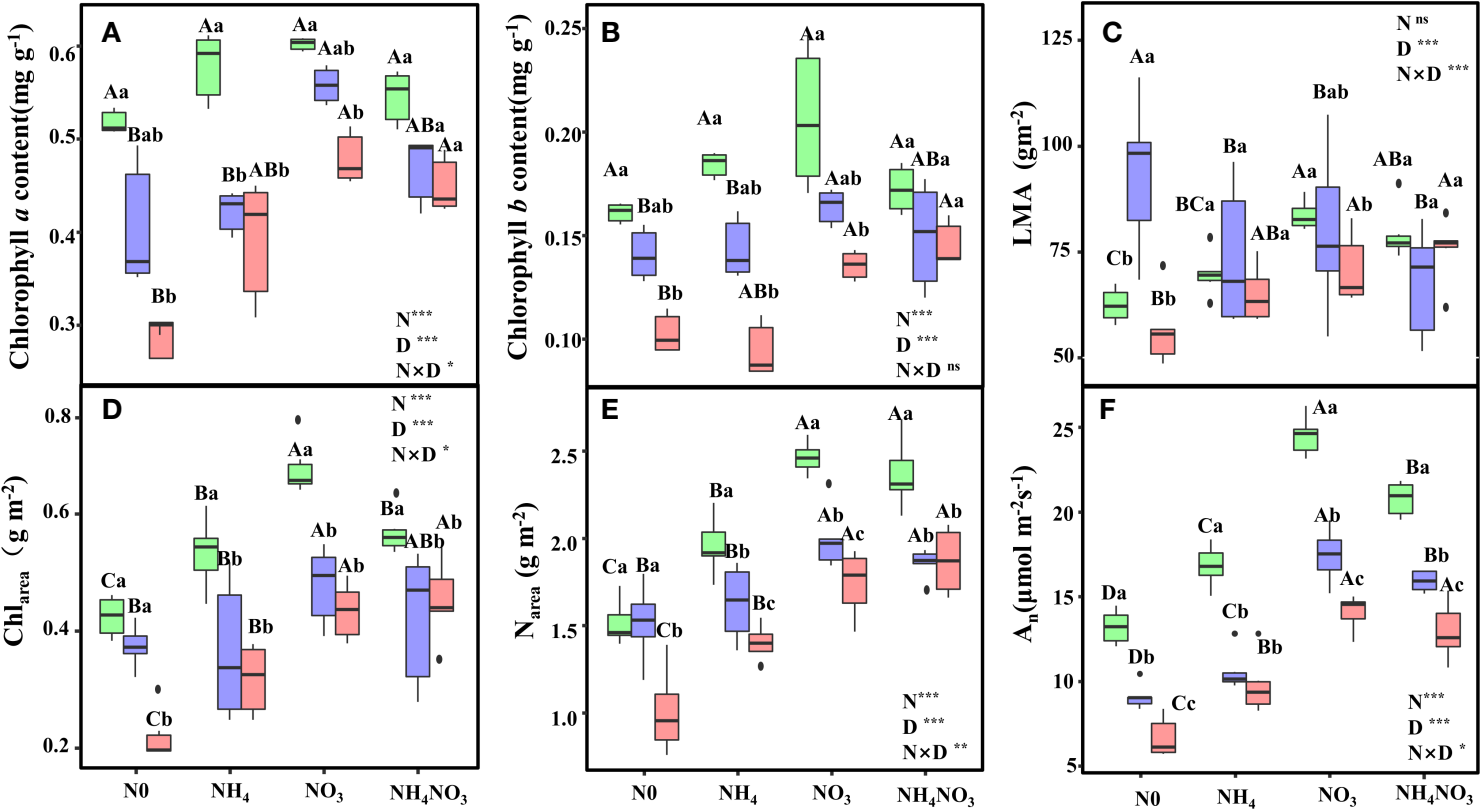
Effects of different nitrogen forms and drought stress on Chlorophyll *a* content **(A)**, Chlorophyll *b* content **(B)**, a leaf mass per unit leaf area (LMA) **(C)**, area-based chlorophyll content (Chl_area_) **(D)**, area-based nitrogen content (N_area_) **(E)**, and the net photosynthetic rate (A_n_) **(F)**. Black dot is “Outlier”; horizontal is “Median”; the top of vertical line is “Max” and the bottom of vertical line is “Min”. Different uppercase letters indicate significant differences under different nitrogen forms (N0, NH_4_, NO_3_ and NH_4_NO_3_) treatments, different lowercase letters indicate significant differences under drought stress (LD, MD, HD) treatments (*P*< 0.05 & n = 6). * indicate p< 0.05; *** indicate p< 0.001.

Significant variations were observed in chlorophyll content due to the combined effects of different N forms and drought stress. Under moderate (MD) and heavy drought (HD) conditions, chlorophyll *a* (Chl*a*) and chlorophyll *b* (Chl*b*) contents in *L. chinensis* leaves were notably higher in plants treated with nitrate (NO_3_) and a mix of ammonium nitrate (NH_4_NO_3_) compared to those treated with no nitrogen (N0) and ammonium (NH_4_) (**Figures 1A, B**, p< 0.05). Leaf mass per unit area (LMA) showed no significant difference between NO_3_ and NH_4_NO_3_ plants under HD, but it was significantly greater than in N0 plants (**Figure 1C**, p< 0.05). Under light drought (LD), the chlorophyll content per unit area (Chl_area_) in leaves was significantly elevated in NO_3_ plants relative to N0, NH_4_, and NH_4_NO_3_ plants (p< 0.05), showing increases of 59.46%, 26.87%, and 19.21%, respectively. Under MD and HD conditions, Chl_area_ remained significantly higher in NO_3_ and NH_4_NO_3_ plants compared to N0 and NH_4_ plants (**Figure 1D**, p< 0.05). Under HD, nitrogen content per unit area (N_area_) in NO_3_ plants was 73.61% and 25.70% higher than in N0 and NH_4_ plants, respectively (**Figure 1E**, p< 0.05). Across all drought treatments (LD, MD, HD), the leaf assimilation rate (An) was significantly elevated in NO_3_-treated plants compared to N0, NH_4_, and NH_4_NO_3_ treatments (**Figure 1F**, p< 0.05). Particularly under HD, An in NO_3_-treated plants was 111.49%, 44.82%, and 8.84% higher than in N0, NH_4_, and NH_4_NO_3_-treated plants, respectively.

### Effects of different N forms on reactive oxygen species accumulation under drought stress

**Figure 2:**
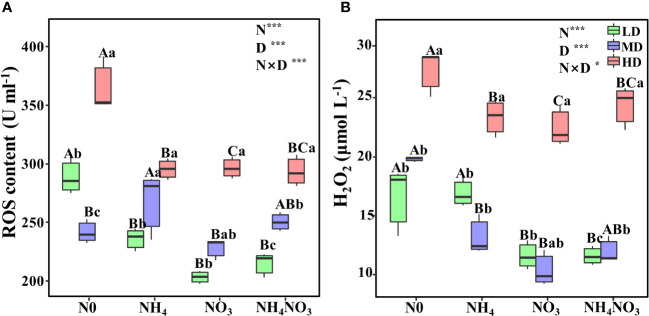
Effects of different nitrogen forms and drought stress on reactive oxygen (ROS) **(A)**, and hydrogen peroxide (H_2_O_2_) **(B)**. Black dot is “Outlier”; horizontal is “Median”; the top of vertical line is “Max” and the bottom of vertical line is “Min”. Different uppercase letters indicate significant differences under different nitrogen forms (N0, NH_4_, NO_3_ and NH_4_NO_3_) treatments, different lowercase letters indicate significant differences under drought stress (LD, MD, HD) treatments (*P*< 0.05 & n = 6). * indicate p< 0.05; *** indicate p< 0.001.

Drought stress triggers an excessive build-up of reactive oxygen species (ROS) and hydrogen peroxide (H_2_O_2_) in *L. chinensis* leaves. Under moderate (MD) and heavy drought (HD) conditions, the ROS levels in plants treated with ammonium (NH_4_), nitrate (NO_3_), and a combination of both (NH_4_NO_3_) were considerably lower than those in plants without nitrogen supplementation (N0). The highest ROS accumulation, reaching 364.75 μmol L^-1^, was observed in N0-treated *L. chinensis* leaves (**Figure 2A**, p< 0.05). As the severity of drought stress increases, so does the accumulation of H_2_O_2_. Under light drought (LD), the H_2_O_2_ content in NO_3_-treated plants was just 10.39 U ml^-1^. However, under MD and HD conditions, the H_2_O_2_ levels in N0 plants rose to 27.31 U ml^-1^, significantly surpassing those in NH_4_, NO_3_, and NH_4_NO_3_ plants (**Figure 2B**, p< 0.05).

### Different N forms induce changes in photoinhibition of photosystem II under drought stress

**Figure 3:**
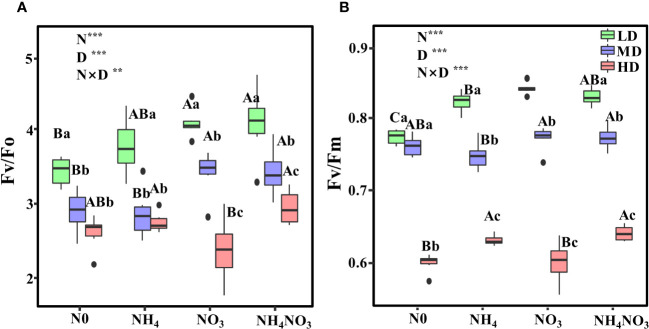
Effects of different nitrogen forms and drought stress on potential activity of PSII (Fv/Fo) **(A)**, the maximum quantum yield of PSII (Fv/Fm) **(B)**. Black dot is “Outlier”; horizontal is “Median”; the top of vertical line is “Max” and the bottom of vertical line is “Min”. Different uppercase letters indicate significant differences under different nitrogen forms (N0, NH_4_, NO_3_ and NH_4_NO_3_) treatments, different lowercase letters indicate significant differences under drought stress (LD, MD, HD) treatments (*P*< 0.05 & n = 6). ** indicate p< 0.01; *** indicate p< 0.001.

Different N forms and drought stress exert a significant effect on the potential activity of PSII (Fv/Fo) and the maximum photochemical efficiency of photosystem II (Fv/Fm) (p< 0.01). Both Fv/Fo and Fv/Fm values declined in leaves subjected to MD and HD drought conditions (**Figure 3**). In NH_4_NO_3_-treated plants, Fv/Fo decreased by 16% under MD and by 27.7% under HD-treated plants compared to LD-treated plants (**Figure 3A**; p< 0.05). For NO_3_-grown plants, Fv/Fm experienced a reduction of 8.2% under MD and 28.6% under HD (**Figure 3B**), while in NH_4_NO_3_-treated plants, the decreases were 6.7% and 22.73% under MD and HD, respectively (**Figure 3B**).

### Different N forms induce changes in quantum efficiency of photosystem II under drought stress

**Figure 4:**
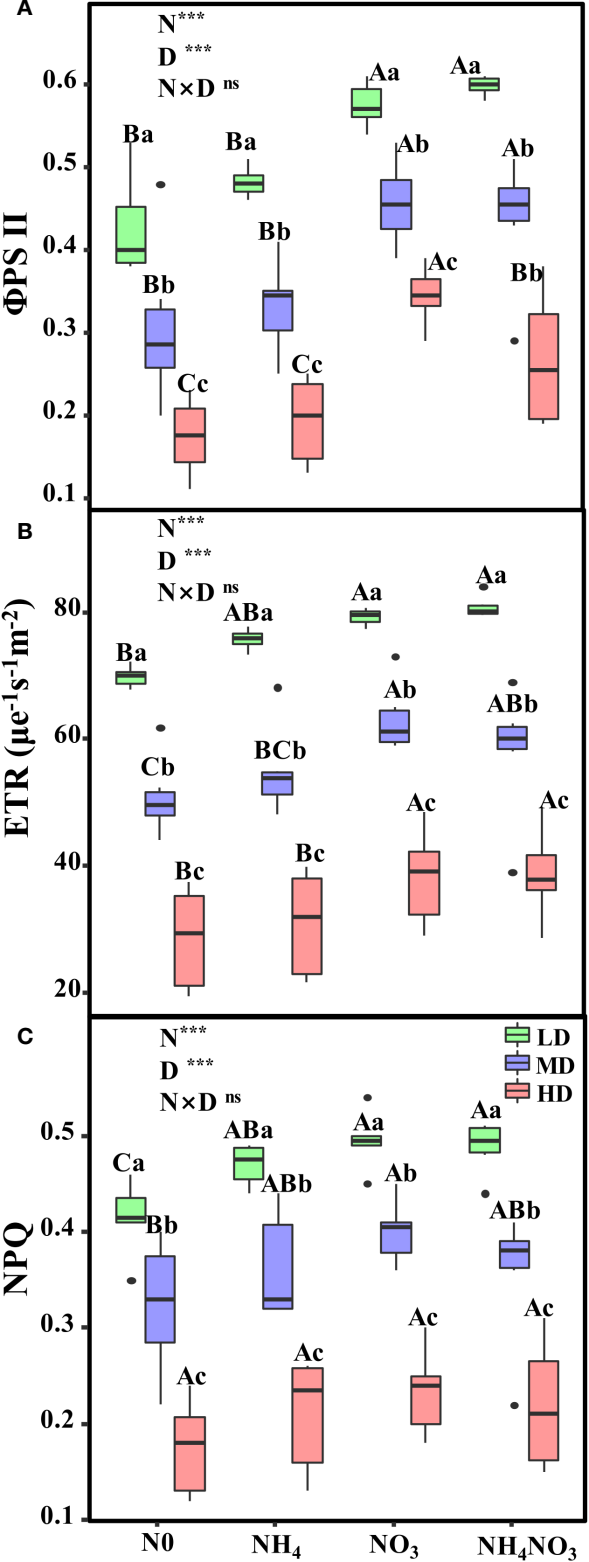
Effects of different nitrogen forms and drought stress on the effective quantum yield of PSII (φPSII) **(A)**, the electron transport rate (ETR, μmol e^-1^ s^-1^ m^-2^) **(B)**, and the nonphotochemical quenching coefficient (NPQ) **(C)**. Black dot is “Outlier”; horizontal is “Median”; the top of vertical line is “Max” and the bottom of vertical line is “Min”. Different uppercase letters indicate significant differences under different nitrogen forms (N0, NH_4_, NO_3_ and NH_4_NO_3_) treatments, different lowercase letters indicate significant differences under drought stress (LD, MD, HD) treatments (*P*< 0.05 & n = 6). *** indicate p< 0.001.

Significant variations were observed in the effective quantum yield of PSII (φPSII), the electron transport rate of PSII (ETR), and the non-photochemical quenching coefficient (NPQ) in plants subjected to moderate (MD) and heavy drought (HD) treatments (**Figure 4**, p< 0.05). Both φPSII and ETR were considerably higher in plants grown with nitrate (NO_3_) and a mixture of ammonium nitrate (NH_4_NO_3_) under MD and HD conditions (**Figures 4A, B**; p< 0.05). In NO_3_ and NH_4_NO_3_ plants under HD stress, φPSII values decreased by 40.2% and 55.4%, respectively (**Figure 4A**). Similarly, ETR values in NO_3_ and NH_4_NO_3_ plants under HD diminished by 51.8% and 52.1%, respectively (**Figure 4B**). However, HD-treated plants showed no significant change in NPQ when treated with NH_4_, NO_3_, and NH_4_NO_3_, with reductions of 55.5%, 52.8%, and 55.5% respectively (**Figure 4C**).

### Responses of the O-J-I-P kinetic curve to drought stress under N forms

**Figure 5:**
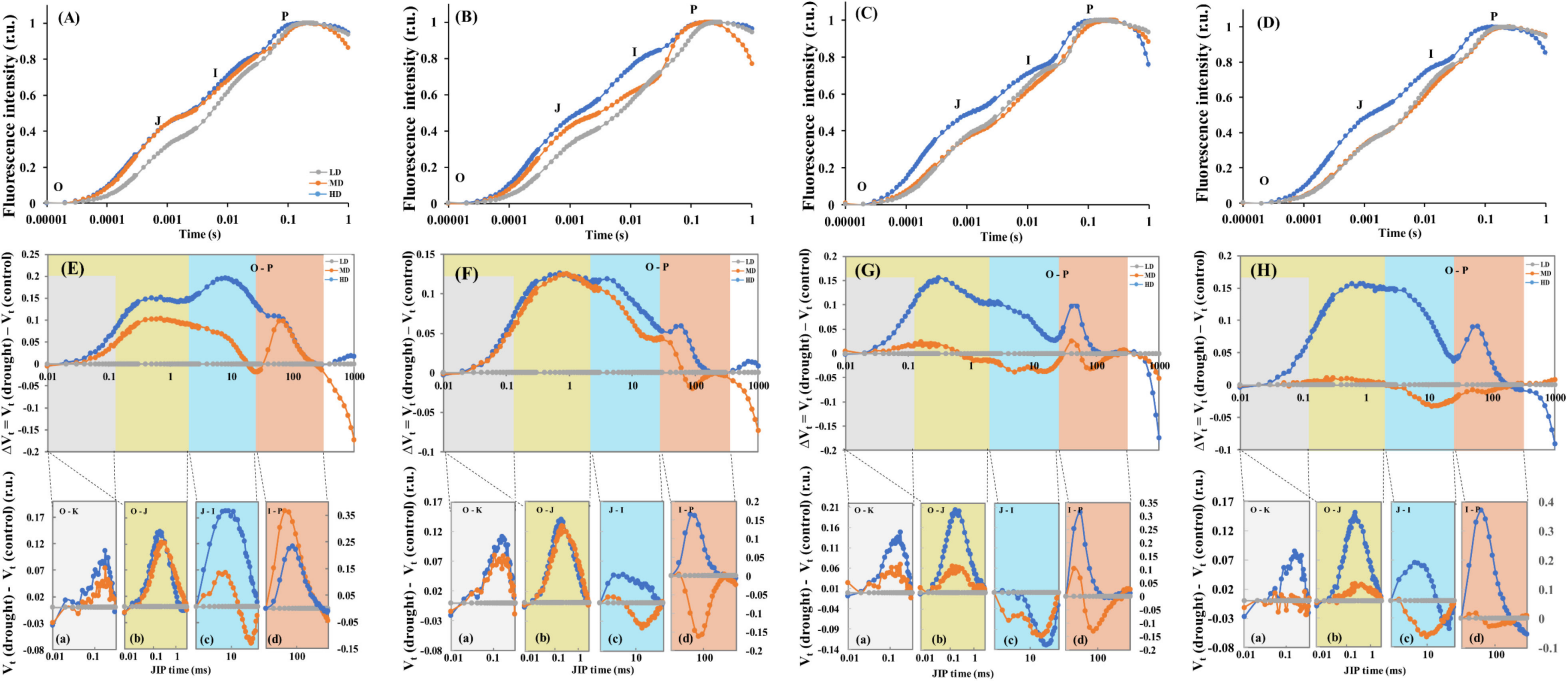
Response of chlorophyll a fluorescence transient (O-J-I-P) curves and differential curves showing differences between *L. chinensis* leaves under different nitrogen forms from drought stress and control treatments. N0 treatment **(A, E)**, NH_4_ treatment **(B, F)**, NO_3_ treatment **(C, G)**, and NH_4_NO_3_ treatment **(D, H)**. Drought stress treatments (LD, MD, HD). Each DC value was calculated as a difference between the values of the relative variable fluorescence [V_t_ = (F_t_– F_O_)/(F_M_– F_O_)] recorded in *L. chinensis* leaves of the MD and HD treatments minus the respective values for control treatment, respectively [ΔV_t_ = V_t (Drought)_ – V_t (Control)_]. The four characteristic bands are marked with different colors **(A-D)**, and L band (a), K band (b), H band (c) and G band (d) show details in each band. For panels **(A-D)** the values for the curves are related to the left scale of Y-axes, for panel d to the right scale.

Under moderate (MD) and heavy drought (HD) treatments, the ΔV_OJ_ and ΔV_OI_ values in *L. chinensis* leaves treated with no nitrogen (N0) increased, with ΔV_IP_ also rising under HD conditions (**Figure 5A**). In leaves treated with ammonium (NH_4_), ΔV_OI_ exhibited a decreasing trend under MD treatment (**Figure 5B**). Drought stress led to noticeable relative disaggregation in the antenna complex within the L-band of *L. chinensis* leaves (**Figures 5E**-a, **F**-a). Under drought, leaves showed positive differences in the K-band curve, with similar peak values under both MD and HD conditions (**Figures 5E**-b, **F**-b). The H-band under HD treatment indicated positive differences, while it shifted from positive to negative under MD treatment (**Figure 5E**-c). Positive differences were observed in the G-band for both MD and HD treatments, with higher peak values under MD (**Figure 5E**-d). Under HD treatment, the H-band displayed positive differences, whereas under MD treatment, it showed negative differences (**Figure 5F**-c). The G-band under HD treatment had positive differences, but under MD treatment, the peak values were negatively different (**Figure 5F**-d). For leaves treated with NO_3_ and NH_4_NO_3_, ΔV_OJ_, ΔV_OK_, and ΔV_OI_ showed increasing trends during the O-J-I-P transition under HD treatment, and ΔV_IP_ also increased (**Figures 5C, D**). Both HD and MD treatments caused positive differences in the L-band, with higher peak values under HD (**Figures 5G**-a, **H**-a). Under drought, NO_3_-treated leaves displayed positive differences in the K-band (**Figure 5G**-b), while NH_4_NO_3_-treated leaves also showed positive differences (**Figure 5H**-b). In NO_3_-treated leaves, the H-band had negative differences under drought (**Figure 5G**-c), but in NH_4_NO_3_-treated leaves, it showed positive differences (**Figure 5H**-c). The early peak of the G-band under HD treatment exhibited positive differences, with later peak amplitude decreasing (**Figure 5G**-d), whereas the G-band in NH_4_NO_3_-treated leaves tended toward positive differences (**Figure 5H**-d).

### Different N forms induce changes in chlorophyll a fluorescence under drought stress

**Figure 6:**
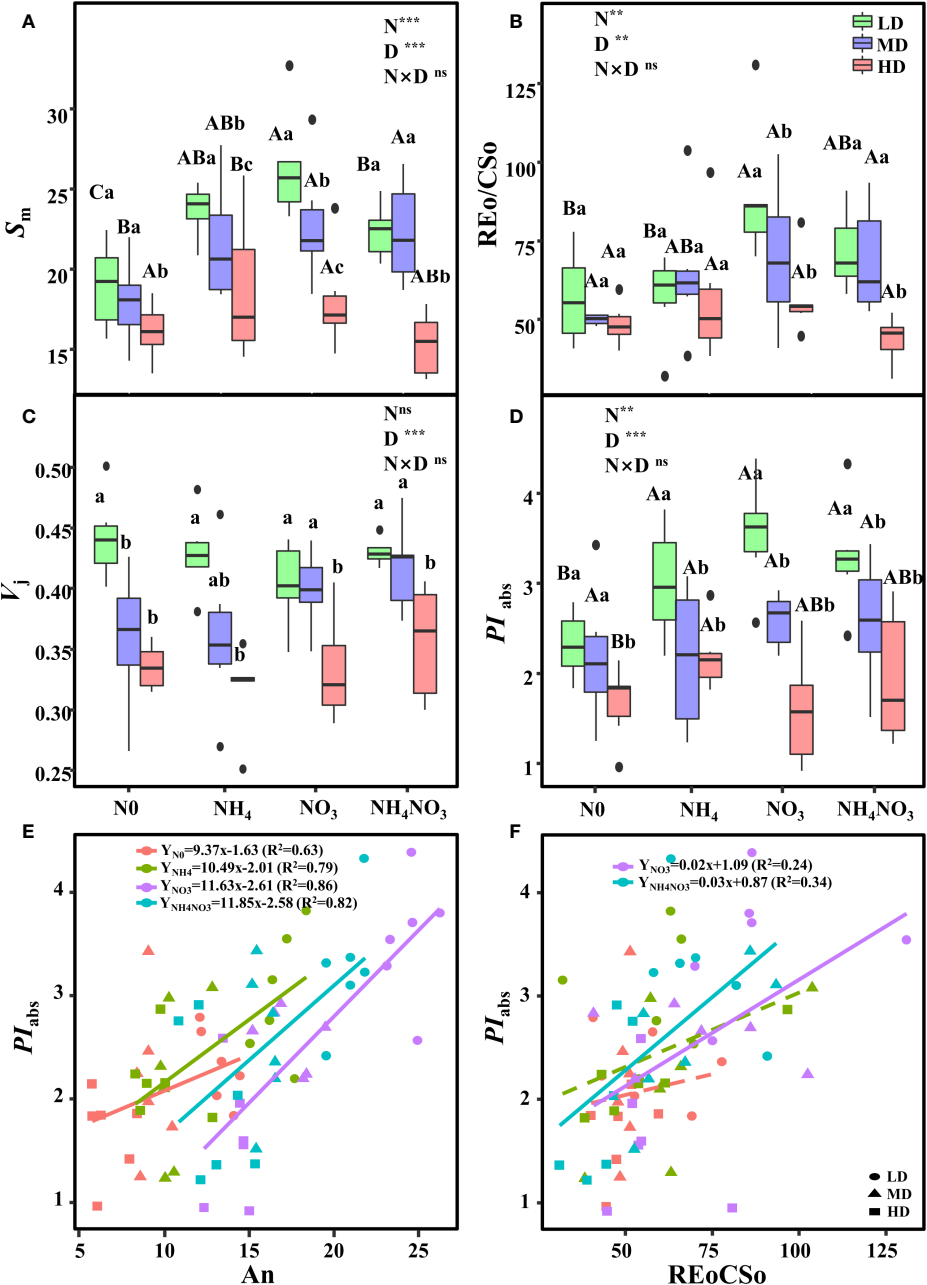
Effects of different nitrogen forms and drought stress on the O-J-I-P transient (reflecting multiple-turnover Q_A_ reduction events) (*S*_m_) **(A)**, reduction of end acceptors at PSI electron acceptor side per CS (at t=0) (REo/CSo) **(B)**, relative variable fluorescence at J- step (*V*_j_) **(C)**, and the performance index on absorption basis (*PI*_abs_) **(D)**. Black dot is “Outlier”; horizontal is “Median”; the top of vertical line is “Max” and the bottom of vertical line is “Min”. Figures represent the correlation between A_n_ and *PI*_abs_ **(E)**, Figures represent the correlation between REo/CSo and *PI*_abs_ **(F)**. Different uppercase letters indicate significant differences under different nitrogen forms (N0, NH_4_, NO_3_ and NH_4_NO_3_) treatments, different lowercase letters indicate significant differences under drought stress (LD, MD, HD) treatments (*P*< 0.05 & n = 6). ** indicate p< 0.01; *** indicate p< 0.001.

Further analysis of the JIP-test parameters revealed that drought stress significantly impacted the photosynthetic characteristics of PSII after the application of NO_3_ and NH_4_NO_3_ (**Figure 6**; p< 0.05; **Supplementary Figure 1**). S_m_ (indicating multiple-turnover Q_A_ reduction events), *V*_j_ (relative variable fluorescence at the J-step), and REo/CSo (Reduction of end acceptors at PSI electron acceptor side per CS at t=0) decreased in N0 and NH_4_-grown plants treated with MD and HD (**Figures 6A, B**). However, *V*_j_ (relative variable fluorescence at the J-step) showed no significant difference in plants treated with different N forms under HD (**Figure 6C**). *PI*_abs_ (performance index for energy conservation from photons absorbed by PSII antenna to the reduction of Q_B_) decreased in N0-grown plants treated with LD and HD (**Figures 6D**). NO_3_-grown plants treated with HD exhibited significantly higher S_m_ and REo/CSo (**Figures 6A, B**; p< 0.05).

The correlation analysis indicated a strong correlation between *PI*_abs_ and An with respect to N forms. To further evaluate the N forms supplied to *L. chinensis*, a model based on the parameters *PI*_abs_ and An was developed. The *PI*_abs_ values increased linearly with the An level, revealing a significant positive linear correlation under NO_3_ treatment (R^2 = ^0.86) (**Figure 6E**). To elucidate the improvement mechanism of photosynthesis in NO_3_-grown plants treated with HD, the relationship between REo/CSo and *PI*_abs_ was analyzed. The correlation analyses revealed highly active relationships between REo/CSo and *PI*_abs_ in NO_3_ and NH_4_NO_3_-grown plants (**Figure 6F**). This suggests that the up-regulation of PSII overall activity is primarily due to the improved energetic connectivity of PSII units and OEC state, as well as the increased PSII electron transport efficiency in NO_3_-treated plants under drought stress. Clearly, NO_3_^-^ contributes to *L. chinensis* plants coping with drought stress by enhancing photosynthetic capacity.

**Figure 7:**
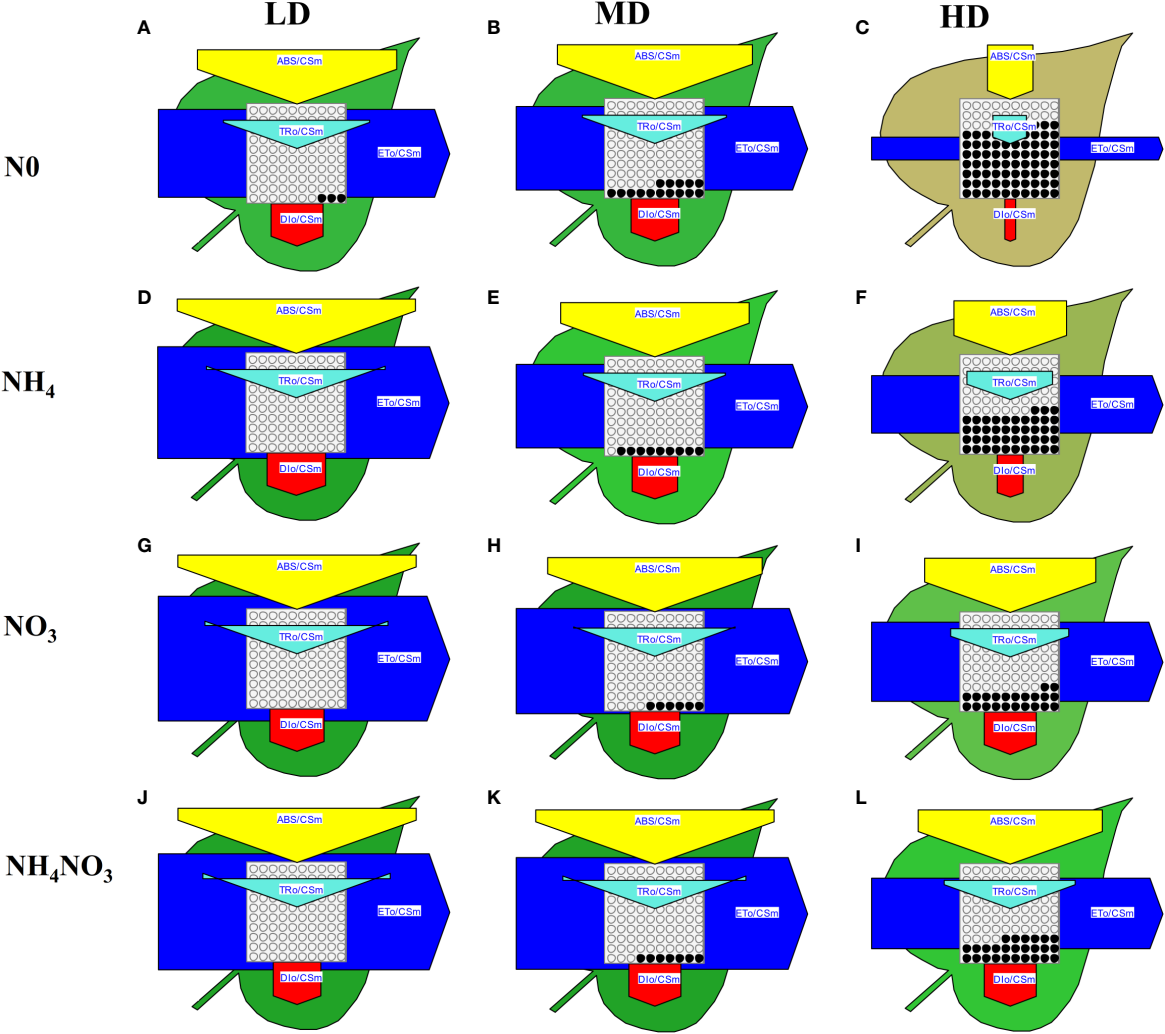
The pipeline models for phenomenological fluxes (leaf model) of *L. chinensis* under different nitrogen forms and drought stress. **(A-L)**. The pipeline models for phenomenological fluxes (leaf model) under drought stress (LD, MD and HD) treatments and different nitrogen forms (N0, NH_4_, NO_3_ and NH_4_NO_3_) treatments. Each arrow thickness represents the values of electron transport flux (ETo/CSm) (blue pentagon), absorbance (ABS/CSm) (yellow pentagon), heat dissipation of excess light (DIo/CSm) (red pentagon), active/inactive reaction centers ascircles inscribed in the aquare (white: active, black: inactive) (RC/CSm) and trapping energy flux (TRo/CSm) (light green triangle); all expressed per leaf CS (green leaf), respectively.

The phenomenological models of energy fluxes through the cross sections (CS) of the leaves of *L. chinensis* under different N forms and drought stress are presented in **Figure 7** and **Supplementary Figure 2**. Deprivation of N0 or NH_4_ significantly diminished the energy fluxes in the plants compared to those supplied with NO_3_ or NH_4_NO_3_ under drought stress. Additionally, NO_3_ or NH_4_NO_3_ resulted in a significant decrease in the energy absorption by a cross section of the leaves (ABS/CS), energy trapping (TR/CS), the electron transport flux (ET/CS), and energy dissipation (DI/CS) under HD compared with LD treated, but the decrease in all the aforementioned parameters was significantly lower than those observed under N0 and NH_4_ supplied (**Figures 7C, F, I, L**). Under HD treated, the TRo/RC increased in NO_3_ and NH_4_NO_3_ treatments, with TRo/RC changes resembling those of ABS/RC; ETo/RC increased in N0 and NO_3_ treatments and decreased in NH_4_ and NH_4_NO_3_ treatments; DIo/RC decreased in NH_4_ treatment, and significantly increased in NO_3_ and NH_4_NO_3_ treatments (**Supplementary Figures 2C, F, I, L**).

### Different N forms induce changes in photosynthetic apparatus enzyme activity under drought stress

**Figure 8:**
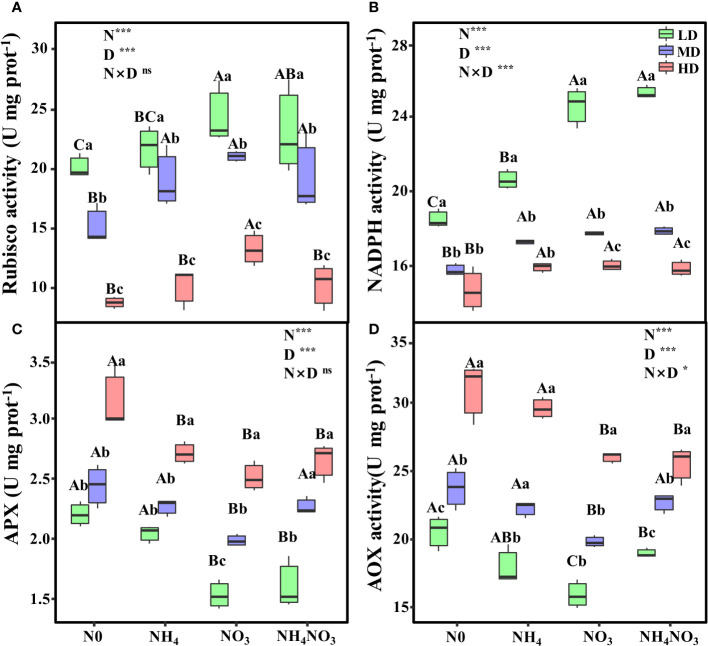
Effects of different nitrogen forms and drought stress on ribulose-1,5-bisphosphate carboxylase/oxygenase (Rubisco) **(A)**, nicotinamide adenine dinucleotide phosphate (NADPH) **(B)**, ascorbate peroxidase (APX) **(C)**, and alternative oxidase (AOX) activity **(D)**. Black dot is “Outlier”; horizontal is “Median”; the top of vertical line is “Max” and the bottom of vertical line is “Min”. Different uppercase letters indicate significant differences under different nitrogen forms (N0, NH_4_, NO_3_ and NH_4_NO_3_) treatments, different lowercase letters indicate significant differences under drought stress (LD, MD, HD) treatments (*P*< 0.05 & n = 6). *indicate p< 0.05; *** indicate p< 0.001; * indicate p< 0.05; *** indicate p< 0.001..

The interaction between different N forms and drought stress profoundly influences the activities of Rubisco, NADPH, and AOX. Notably, under HD treatment, Rubisco activity in NO_3_ plants exhibited a remarkable increase, surpassing N0, NH_4_, and NH_4_NO_3_ plants by 51.27%, 31.01%, and 29.64%, respectively (**Figure 8A**, p< 0.05). Moreover, during MD and HD treatments, NADPH and APX activities in NH_4_, NO_3_, and NH_4_NO_3_ plants, while not significantly different from each other, were significantly higher compared to N0 plants (**Figures 8B, C**, p< 0.05). Interestingly, under HD treatment, AOX activity in NO_3_ and NH_4_NO_3_ treated plants significantly decreased that in N0 and NH_4_ plants (**Figure 8D**, p< 0.05).

## Discussion

Drought stress is an abiotic stress factor that can influence plant growth while photosynthesis serves as the primary energy source for plant growth and an important pathway for increasing yield. Photosynthesis is highly sensitive to soil drought stress, because such stress can cause a reduction in photosynthetic enzyme activity, disintegration of the thylakoid membranes, and the degradation of photosynthetic proteins, thereby inhibiting photosynthetic capacity. In the present study, we investigated the impact of electron transfer on PSII photoinhibition in the dominant species *L. chinensis* under drought stress. When compared to treatments with NO_3_ and NH_4_NO_3_, plants treated with N0 and NH_4_ exhibited significantly inhibited PSII activity under drought stress. Our current research revealed the mechanisms responsible for drought-induced PSII photoinhibition in *L. chinensis* subjected to different forms of N.

### Nitrate nitrogen alleviates drought damage to *L. chinensis* plants by maintaining high photosynthesis

N plays a pivotal role in shaping key components like Rubisco, chlorophyll, and photosynthetic proteins, underscoring its significance in plant photosynthesis (Li et al., 2013; Mu and Chen, 2021). The efficient absorption and utilization of N can profoundly impact the photosynthetic N use efficiency of plants, thereby influencing overall photosynthetic efficiency (Evans and Clarke, 2019). Existing evidence indicates a positive correlation between the photosynthetic efficiency of *L. chinensis* and increasing photosynthetic N use efficiency (PNUE) (Wei et al., 2022). Photosynthesis is intricately linked to the N and chlorophyll contents in leaves (Rogers et al., 2020). Elevated levels of N_area_ and Chl_area_ have been associated with enhanced light capture (Hikosaka, 2004). Our findings align with previous research (Liang et al., 2020), demonstrating that under drought conditions, N0 and NH_4_ treatments led to reduced LMA, chlorophyll, and N_area_, resulting in diminished light capture and suppressed photosynthesis (**Figure 1**). Consequently, maintaining higher chlorophyll content through the application of NO_3_ and NH_4_NO_3_ treatments proved effective in sustaining the photosynthetic performance of leaves under drought stress.

### Nitrate nitrogen enhances photosynthesis of *L. chinensis* plants by improving the PSII electron transport efficiency

Plants can also utilize the NPQ mechanism to dissipate an excess of absorbed light energy under sufficient sunlight, thereby avoiding the formation of ROS that can exert detrimental effects on the photosynthetic apparatus (Souza et al., 2022). When the light energy absorbed by the antenna pigments exceeds the capacity of the photosynthetic apparatus, then photochemical inhibition occurs in both PSI and PSII (Sonoike, 2011). Furthermore, it has been reported that *E. adenophorum* (Spreng.) and *P. notoginseng* (Burkill.) enhance electron transport to adapt to fluctuations in light and improve the efficiency of light energy utilization (Feng et al., 2009; Cun et al., 2022). In the present study, we also analyzed the impact of drought stress on the PSII reaction centers in *L. chinensis* leaves and found that in plants treated with NO_3_ and NH_4_NO_3_, Fv/Fm decreased to a lesser extent under drought stress. The reduction in leaf pigment content during drought stress did not significantly reduce Fv/Fo and Fv/Fm (**Figure 3**). These results suggest that the changes in leaf pigment content had a minimal impact on Fv/Fm, thus indicating that the reduction in pigment synthesis may be a pathway by which plants reduce light absorption to establish photoprotection mechanisms (Galmés et al., 2007). In this study, we observed significant differences in Fv/Fm in the presence of different N forms and under drought stress conditions, thus indicating that Fv/Fm is more sensitive to drought stress when subjected to different N treatments. φPSII, ETR and NPQ data all showed that plants treated with NO_3_ and NH_4_NO_3_ exhibited a smaller reduction under drought stress (**Figure 4**). We speculate that the reduction in PSII activity during PSII photoinhibition leads to a reduction in ETR. Similar findings have also been reported in *L. chinensis* plants grown under N0 and NH_4_ conditions (Zhong et al., 2019; Wei et al., 2022).

### Nitrate nitrogen enhances photosynthesis of *L. chinensis* plants by improving the OEC state and energetic connectivity of PSII units

Under drought stress, the PSII reaction centers can undergo reversible inactivation, where they can absorb light energy but do not transfer the absorbed energy to the electron transport chain (Banks, 2018). When the donor side of PSII is damaged, there are changes in chlorophyll a fluorescence in the O-K interval (within a very short time in the L band), thus reflecting the grouping level of PSII within thylakoid membranes and the probability of redistribution of excitation energy between them (**Figure 5**). The movement of PSII antenna complexes can occur, and any changes in these structures during the growth and development of plants can result in changes in the efficiency of interaction between antenna complexes, thus influencing energy transfer within the process of vegetation growth. Changes in the L band (50-300 μs) can reveal energy transfer between PSII antenna complexes (Strasser et al., 2000; Oukarroum and Strasser, 2004; Strauss et al., 2006). Under drought stress and various N treatments, the difference in the L band was positive, with smaller variations for NO_3_ and NH_4_NO_3_ treatments, thus indicating only mild damage to the leaves of *L. chinensis* plants. The K band (5-20 ms) reflects the photochemical reduction of Q_A_^-^ and the partial re-oxidation of Q_A_^-^ by PQ *via* Q_B_^-^ (Tsimilli-Michael and Strasser, 2008). Changes in the K band at approximately 300 μs directly reflect changes in the oxygen-evolving complex (OEC), especially the Mn-complex on the donor side of PSII (Strasser, 1997; Yusuf et al., 2010), thus indicating the functionality of electron transfer on the donor and acceptor sides of PSII. Under HD and MD drought stress treatments, the difference in the K band was positive for all N treatments, although the trends of difference for plants treated with NO_3_ and NH_4_NO_3_ were lower. This is because electron transfer from the donor side was relatively fast, the OEC was not completely inactivated, and electron withdrawal from the acceptor side was accelerated, thereby alleviating damage to the donor side under drought stress (Guha et al., 2013). The H band (2-30 ms) reflects the dynamic process of a reduction in the PQ pool between two chlorophyll fluorescence sites (Harley et al., 1992; Strasser et al., 2010). Under drought stress, the H band was negative for the NO_3_ treatment; these finding suggest that under drought stress, the NO_3_ treated leaves were beneficial for increasing the PQ pool, promoting proton or electron transfer, and further facilitating the transfer of energy and substances. Changes in the G band (30-300 ms) reflect a reduction in the electron acceptor pool at the PSI terminal, and the rate of decrease determines the variation in the peak of the G band. As the electron acceptor pool increases, the rate of reaching the maximum value slows down, thus resulting in a transient negative value in the G band. When the electron acceptor pool at the PSI terminal decreases, the transient rate accelerates, thus resulting in a positive peak in the differential fluorescence curve (Küpper et al., 2019). Under drought stress, leaves reduce the relative proportion of the more accessible acceptor, and this reduction accelerates, thus resulting in a positive peak in the G band. Conversely, an increase in the more accessible acceptor leads to a negative value. We found that the application of NO_3_^-^-N and NO_3_^-^-N: NH_4_^+^-N slowed down the reduction in the more accessible acceptors.

In the N0 and NH_4_ treatments, drought stress increased the flow of electrons from the leaf area to the PSI end (REo/CSo); however, due to the restriction of photosynthetic electron transfer, the amount of light energy transferred from the reaction centers to PSI (REo/RC) also increases, thus resulting in a reduced capacity of the leaf to consume excess light energy. However, under the NO_3_ and NH_4_NO_3_ treatments, the opposite effect was observed. Under the NO_3_ treatment, the electron acceptor on the acceptor side of PSII (S_m_) increased, thus indicating the enhanced probability of electron transfer from captured light energy to Q_A_^-^ (Strasser et al., 2010). When analyzing *PI*_abs,_ we observed that the application of different N forms modified the response of *L. chinensis* to drought stress (**Figure 6**, **Supplementary Figure 1**). Moreover, *PI*_abs_, identified as the most sensitive parameter in the O-J-I-P kinetic curve, serves as an efficient approach for assessing and determining the resilience of plants to stress (Cun et al., 2022). The absolute value of the slope (K) is 9.37 (N0), 10.49 (NH_4_), 11.63 (NO_3_), and 11.85 (NH_4_NO_3_), respectively (**Figure 6E**). A significant positive linear correlation was observed between *PI*_abs_ and An (**Figure 6E**). It is evident that *L. chinensis* tends to utilize NO_3_^-^ to enhance photosynthetic capacity to tolerate drought stress. This aligns with our previous research results (Wei et al., 2022). Additionally, a significant positive linear correlation was noted between *PI*_abs_ and REoCSo (**Figure 6F**), further confirming that the most crucial determinant of PSII loss of function is the damage to OEC centers (Chen et al., 2016).

Compared to NO_3_^-^-N and the mixture of NH_4_^+^-N: NO_3_^-^-N, the application of NH_4_^+^-N significantly inhibited the growth of *L. chinensis* under drought stress. The leaf electron transport pathway model (also referred to as the phenomenological energy flux model) and the thylakoid membrane model are widely used to analyze the effects of biotic or abiotic stresses on plants (Pan et al., 2018; Faseela et al., 2019). In response to drought stress, plant leaves exhibit a reduction in the absorbed light energy per unit area (ABS/CSm) (Faseela et al., 2019), thus indicating that drought stress may lead to the degradation or inactivation of reaction centers, or changes in the structure or degradation of antenna pigments, thus resulting in a reduction in the captured light energy. In the present study, we found that a reduction in ABS/CSm inhibits the excitation energy used to reduce Q_A_^-^ (TRo/CSm) per unit area, as well as the reduction energy (ETo/CSm) entering the additional electron transfer chain (Wang F. et al., 2019). Furthermore, drought stress led to a reduction in thermal dissipation per unit leaf area (DIo/CSm) and a reduction in the activity of the reaction centers per leaf cross-section (RC/CSm) (Hu et al., 2023). These results indicated that *L. chinensis* leaves activate defense mechanisms when subjected to drought stress, including a reduced leaf area, weakened transpiration, and impeded dissipation of excess excitation energy within the leaf (Guo et al., 2020). We found that the plants treated with NO_3_ and NH_4_NO_3_ were able to alleviate the reduction in ABS/CSm, TRo/CSm, and ETo/CSm (**Figure 7**, **Supplementary Figure 2**). Furthermore, drought stress reduced the number of active reaction centers (RC) per unit leaf area, thereby promoting the efficiency of additional active reaction centers within the leaf, reducing leaf area, and improving the efficiency of excess excitation energy dissipation (Wang B. et al., 2019; Wang F. et al., 2019). In *L. chinensis* leaves treated with NO_3_ and NH_4_NO_3_, the absorbed light energy per active reaction center (ABS/RC) and the excitation energy used to reduce Q_A_^-^ (TRo/RC) increased, thereby increasing thermal dissipation (DIo/RC) (Pandey et al., 2023).

### Nitrate nitrogen alleviates oxidative damage by promoting photosynthetic apparatus enzyme activity in *L. chinensis* plants

During drought stress, the oxidation of Rubisco by RuBP in the photosynthetic carbon oxidation cycle of C_3_ plants forms a major alternative sink of electrons, thus maintaining partial oxidation of the PSII acceptor and preventing PSII photoinactivation when CO_2_ concentrations decrease (Faizan et al., 2023). The activity of the AOX enzyme is known to be closely related to the accumulation of ROS under drought stress, which can be detrimental to plants (Bartoli et al., 2004). Plants are known to induce the generation of ROS when subjected to environmental stress. However, during the process of evolution, plants have developed rapid and appropriate responses to prevent the formation of ROS induced by environmental stress during growth, development, and defense processes (Huang et al., 2021; Mittler et al., 2022). In most C_3_ plants, ROS and H_2_O_2_ are generated by the oxidation of malate in the peroxisome *via* the photosynthetic carbon oxidation cycle pathway. Plants usually activate defense mechanisms, including antioxidant enzymes, photoprotection, and the respiratory electron transport chain, to maintain osmotic pressure and balance energy transfer (Ahanger et al., 2017; Huang et al., 2021). In the present study, the levels of ROS in plants treated with NO_3_ and NH_4_NO_3_ under drought stress were significantly lower than those in *L. chinensis* plants treated with N0 and NH_4_, thus indicating that *L. chinensis* plants treated with N0 and NH4 were more sensitive to drought stress. The application of NO_3_^-^-N and NH_4_NO_3_ activated defense mechanisms in plants and alleviated the cell damage caused by drought stress (**Figure 2**). The AOX pathway can also serve as an antioxidant mechanism for plant tolerance to environmental stress (Li et al., 2021). In the ascorbate-glutathione cycle pathway, APX catalyzes the reaction between ascorbic acid and H_2_O_2_, and NADPH uses electron transfer with glutathione as an intermediary to reduce H_2_O_2_ to H_2_O, thereby eliminating the toxicity caused by excessive levels of H_2_O_2_ (Gupta et al., 2018).

In this study, we analyzed the activity of AOX in plants and found that the AOX activity under NO_3_ and NH_4_NO_3_ treatments was significantly higher than that under N0 and NH_4_ treatments and drought stress (**Figure 8**). Thus, the addition of NO_3_^-^-N and a mixture of NO_3_^-^-N and NH_4_^+^-N can activate AOX, consume the excessive reducing power caused by drought, maintain the electron transport capacity of *L. chinensis* leaves, and reduce oxidative damage. When subjected to drought stress, the application of NO_3_^-^-N and a mixture of NH_4_^+^-N and NO_3_^-^-N enhanced the energy consumption efficiency of the remaining active reaction centers to dissipate energy within the electron transfer chain, thereby mitigating damage to the leaves caused by drought stress. These findings demonstrated that the addition of NO_3_^-^-N optimized the stability of the light-harvesting and electron transfer systems in *L. chinensis* leaves under drought stress, thus maximizing the PSII quantum yield.

## Conclusion

**Figure 9:**
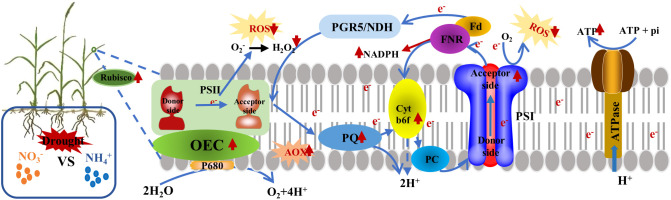
A model proposing the adaptive strategy of *L. chinensis* photosystem under drought stress after the application of nitrate nitrogen was presented. Drought stress inhibits the formation of NADPH and ATP, thereby damaging the donor side of the PSII oxygen-evolving complex (OEC). However, the addition of nitrate nitrogen mitigates the damage rate of PSII under drought stress. Additionally, the accumulation of reactive oxygen species (ROS) is the main cause of PSI over-reduction and PSII photoinhibition in *L. chinensis* under drought stress, while higher rubisco and AOX enzyme activity protect PSII from photodamage. Cracked areas represent damage to the photosystem.

In this study, we demonstrated that drought stress significantly reduced pigment accumulation in the leaves of *L. chinensis*. Different forms of N delayed this inhibitory effect on the photosynthetic electron transfer in leaves, although the extent of this effect varied. Notably, the addition of ammonium nitrogen (NH_4_^+^-N) led to a less pronounced effect in the alleviation of drought stress. In contrast, the application of nitrate nitrogen (NO_3_^-^-N) and a combination of NH_4_^+^-N and NO_3_^-^-N considerably reduced the damage incurred by the photosynthetic machinery in *L. chinensis* under drought conditions, thereby diminishing the growth suppression caused by such stress. This research highlights the existence of a potential adaptation mechanism for *L. chinensis* to drought stress, particularly when treated with NO_3_^–^N. Drought stress triggered an accumulation of ROS and a surge in H_2_O_2_; this disrupts ATP synthesis and damages the donor side of the PSII oxygen-evolving complex (OEC), leading to the over-reduction of the acceptor side of PSI, and consequently, photoinhibition (**Figure 9**). Compared to NH_4_^+^-N, the addition of NO_3_^-^-N reduce the activity of ascorbate peroxidase (APX) and stimulates the alternative oxidase (AOX) pathway. This consumes surplus electrons in the electron transfer chain, alleviates the damage incurred by PSII, reduces photoinhibition in the photosystems, augments the electron transfer rate in *L. chinensis* under drought stress, and ensures the stability of photosynthetic apparatus activity.

## Data availability statement

The original contributions presented in the study are included in the article/**Supplementary Material**. Further inquiries can be directed to the corresponding author.

## Author contributions

XW: Conceptualization, Data curation, Formal analysis, Writing – original draft, Writing – review & editing. LH: Writing – review & editing. NX: Writing – review & editing. MS: Writing – review & editing. XY: Writing – original draft, Writing – review & editing, Funding acquisition, Investigation, Visualization.
