# Supplementary material for: Nitrate nitrogen enhances the efficiency of photoprotection in Leymus chinensis under drought stress
Source: Front Plant Sci. 2024 Feb 14;15:1348925. doi: 10.3389/fpls.2024.1348925 (PMC10899514; doi:10.3389/fpls.2024.1348925)
Supplement: Supplementary file 1 [file DataSheet_1.docx]

**
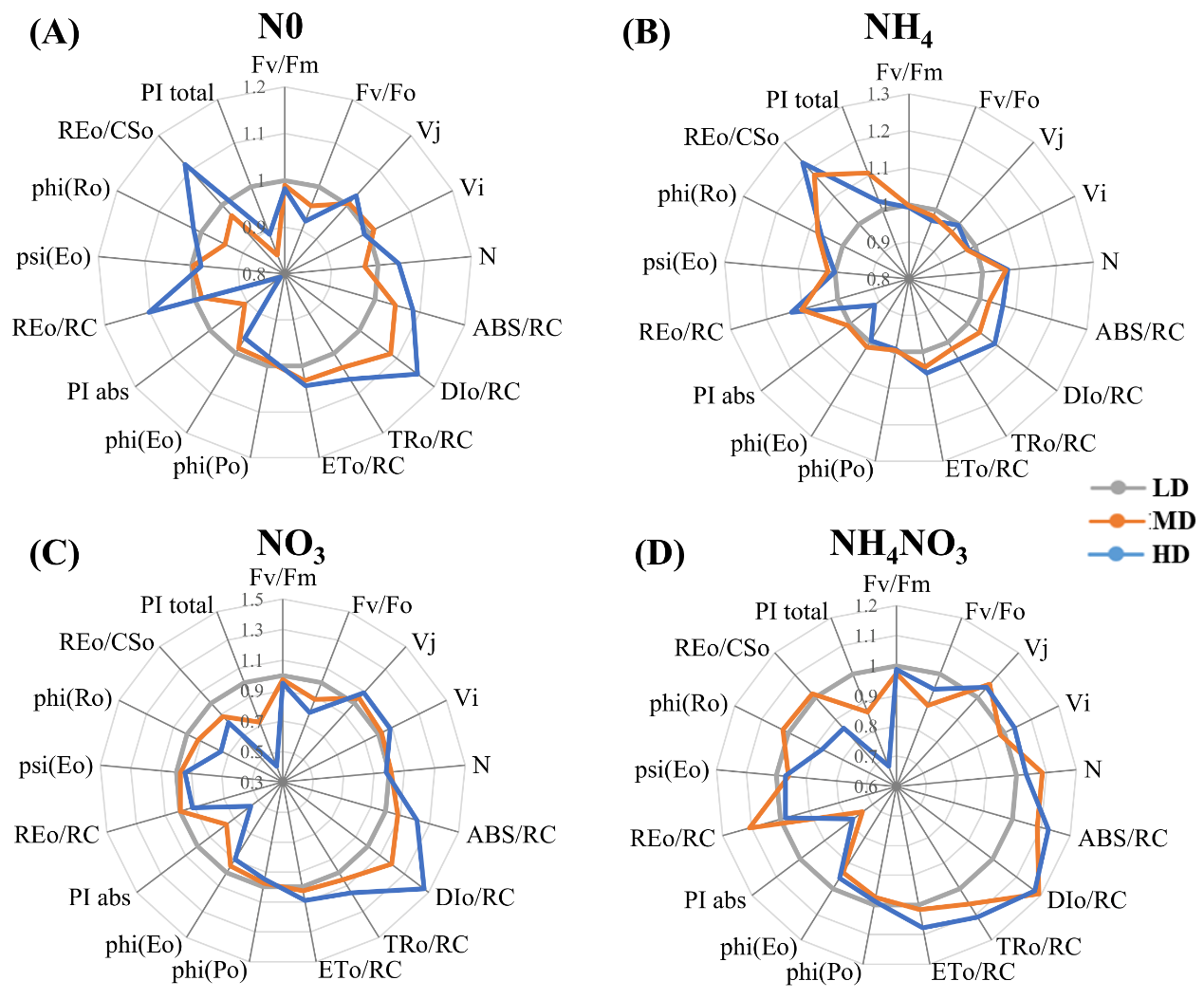
FIGURE S1 |**Radar plot of response of chlorophyll A fluorescence parameters to drought stress under different nitrogen forms of *L. chinensis* leaves. N0 treatment (A), NH_4_ treatment(B), NO_3_ treatment (C), NH_4_NO_3_ treatment (D). Drought stress treatments (LD, MD, HD). The definitions of the abbreviations show in the table of main abbreviations.


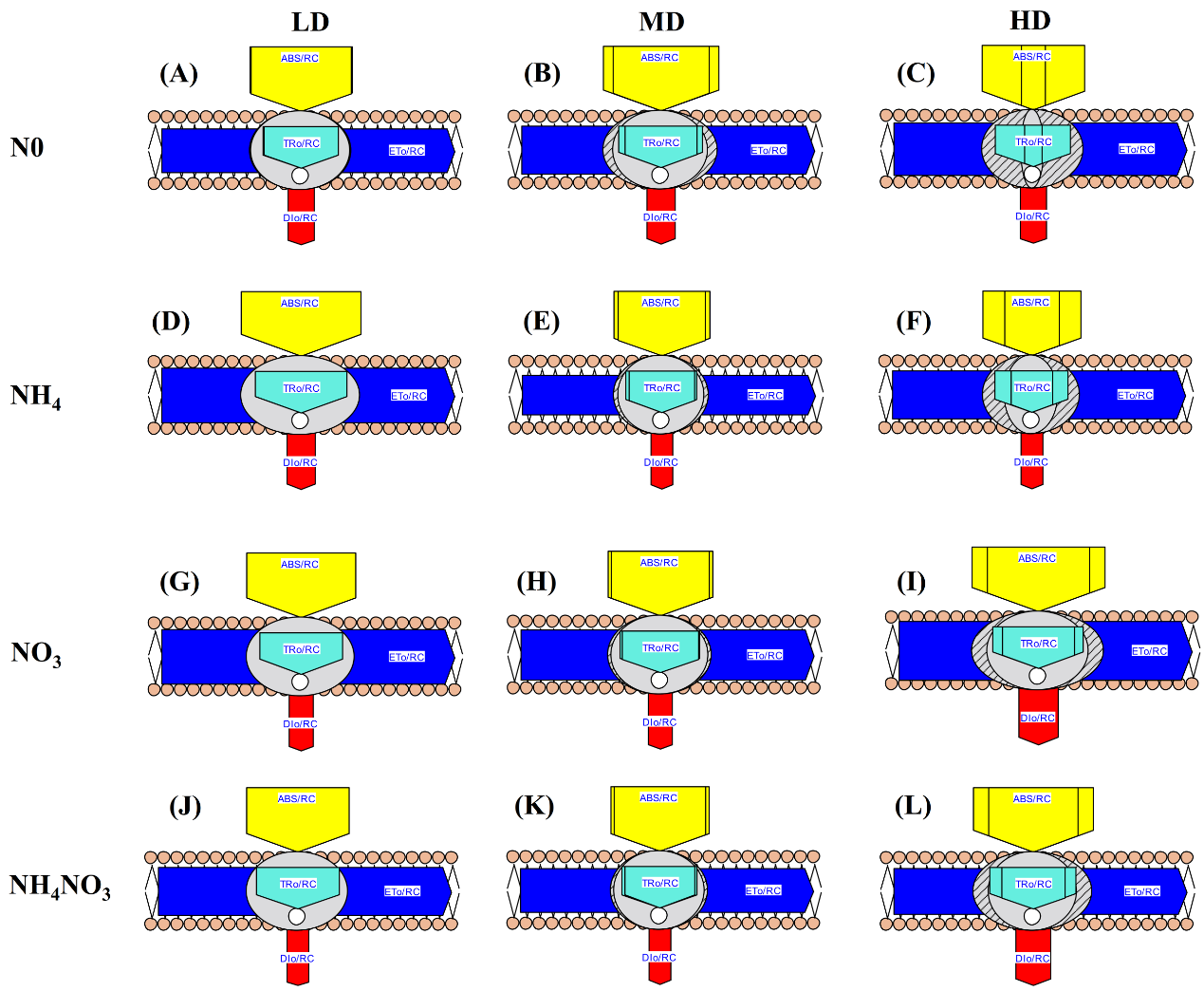


**FIGURE S2 |** The pipeline models for specific fluxes (membrane model) of *L. chinensis* under different nitrogen forms and drought stress. (A)- (L)：The leaf membrane model under drought stress (LD, MD and HD) treatments and different nitrogen forms (N0, NH_4_, NO_3_ and NH_4_NO_3_) treatments. Each arrow thickness represents electron transport flux per RC (ETo/RC) (blue pentagon), absorption flux per RC (ABS/RC) (yellow pentagon), dissipated energy flux per RC (DIo/RC) (red pentagon) and trapped energy flux per RC (TRo/RC) (light green triangle). In membrane models, the gray circles and shadow circles represent the active and inactive RCs, respectively.
